# Parametric sensitivity analysis for biochemical reaction networks based on pathwise information theory

**DOI:** 10.1186/1471-2105-14-311

**Published:** 2013-10-22

**Authors:** Yannis Pantazis, Markos A Katsoulakis, Dionisios G Vlachos

**Affiliations:** 1 Department of Mathematics and Statistics, University of Massachusetts, Amherst, MA 01002, USA; 2 Department of Chemical Engineering, University of Delaware, Newark, Delaware, 19716, USA

**Keywords:** Biochemical reaction networks, Sensitivity analysis, Relative entropy rate, Pathwise Fisher information matrix, p53 model, EGFR model

## Abstract

**Background:**

Stochastic modeling and simulation provide powerful predictive methods for the intrinsic understanding of fundamental mechanisms in complex biochemical networks. Typically, such mathematical models involve networks of coupled jump stochastic processes with a large number of parameters that need to be suitably calibrated against experimental data. In this direction, the parameter sensitivity analysis of reaction networks is an essential mathematical and computational tool, yielding information regarding the robustness and the identifiability of model parameters. However, existing sensitivity analysis approaches such as variants of the finite difference method can have an overwhelming computational cost in models with a high-dimensional parameter space.

**Results:**

We develop a sensitivity analysis methodology suitable for complex stochastic reaction networks with a large number of parameters. The proposed approach is based on Information Theory methods and relies on the quantification of information loss due to parameter perturbations between time-series distributions. For this reason, we need to work on path-space, i.e., the set consisting of all stochastic trajectories, hence the proposed approach is referred to as “pathwise”. The pathwise sensitivity analysis method is realized by employing the rigorously-derived Relative Entropy Rate, which is directly computable from the propensity functions. A key aspect of the method is that an associated pathwise Fisher Information Matrix (FIM) is defined, which in turn constitutes a gradient-free approach to quantifying parameter sensitivities. The structure of the FIM turns out to be block-diagonal, revealing hidden parameter dependencies and sensitivities in reaction networks.

**Conclusions:**

As a gradient-free method, the proposed sensitivity analysis provides a significant advantage when dealing with complex stochastic systems with a large number of parameters. In addition, the knowledge of the structure of the FIM can allow to efficiently address questions on parameter identifiability, estimation and robustness. The proposed method is tested and validated on three biochemical systems, namely: (a) a protein production/degradation model where explicit solutions are available, permitting a careful assessment of the method, (b) the p53 reaction network where quasi-steady stochastic oscillations of the concentrations are observed, and for which continuum approximations (e.g. mean field, stochastic Langevin, etc.) break down due to persistent oscillations between high and low populations, and (c) an Epidermal Growth Factor Receptor model which is an example of a high-dimensional stochastic reaction network with more than 200 reactions and a corresponding number of parameters.

## Background

The need of an intrinsic understanding of the interplay between complexity and robustness of biological processes and their corresponding design principles is well-documented, see for instance [1-5]. The concept of robustness can be described as “a property that allows a system to maintain its functions against internal and external perturbations” [3]. When referring to mathematical models of complex biological processes, one of the mathematical tools to describe the robustness of a system to perturbations is sensitivity analysis which attempts to determine which parameter directions (or their combinations) are the most/least sensitive to perturbations and uncertainty, or to errors resulting from experimental parameter estimation. Recently there has been significant progress in developing sensitivity analysis tools for low-dimensional stochastic processes, modeling well-mixed chemical reactions and biological networks. Some of the mathematical tools included log-likelihood methods and Girsanov transformations [6-8], polynomial chaos [9], finite difference methods and their variants [10,11] and pathwise sensitivity methods [12]. However, existing sensitivity analysis approaches can have an overwhelming computational cost, either due to high variance in the gradient estimators, or in models with a high-dimensional parameter space, [13].

The aforementioned methods focus on the sensitivity of stochastic trajectories and corresponding averages. However, it is often the case that we are interested in the sensitivity of probability density functions (PDF), which are typically non-Gaussian in nonlinear and/or discrete systems. In that latter direction, there is a broad recent literature relying on information theory tools, and where sensitivity is estimated by using the Relative Entropy and the Fisher Information Matrix between PDFs, providing a quantification of information loss along different parameter perturbations. We refer to [14-18] for the case when the parametric PDF is explicitly known. For instance, in [16] the parametric PDF’s structure is known as it is obtained through an entropy maximization subject to constraints. Knowing the form of the PDF allows to carry out calculations such as estimating the relative entropy and identifying the most sensitive parameter combinations. Furthermore, the pathwise PDFs are also known in reaction networks when a Linear Noise Approximation (LNA) is employed and for this case the relative entropy can be explicitly computed allowing thus to carry out parametric sensitivity analysis, [18]. However, for complex stochastic dynamics of large reaction networks, spatial Kinetic Monte Carlo algorithms and molecular dynamics, such explicit formulas for the PDFs are not available in general.

In [19], we address such challenges by introducing a new methodology for complex stochastic dynamics based on the Relative Entropy Rate (RER) which provides a measure of the sensitivity of the entire time-series distribution. Typically, the space of all such time-series is referred in probability theory as the “path space”. RER measures the loss of information per unit time in path space after an arbitrary perturbation of parameter combinations. RER and the corresponding Fisher Information Matrix (FIM) become computationally feasible as they admit explicit formulas which depend only on the propensity functions (see (4) and (6), respectively). In fact, we showed in [19] that the proposed pathwise approach to sensitivity analysis has the following features: First, it is rigorously valid for the sensitivity of long-time, stationary dynamics in path space, including for example bistable, periodic and pulse-like dynamics. Second, it is a gradient-free sensitivity analysis method suitable for high-dimensional parameter spaces as the ones typically arising in complex biochemical networks. Third, the RER method does not require the explicit knowledge of the equilibrium PDFs, relying only on information for local dynamics and thus making it suitable for non-equilibrium steady state systems. In [19], we demonstrated these features by focusing on two classes of problems: Langevin particle systems with either reversible (gradient) or non-reversible (non-gradient) forcing, highlighting the ability of the method to carry out sensitivity analysis in non-equilibrium systems; and spatially extended Kinetic Monte Carlo models, showing that the method can handle high-dimensional problems.

In this paper, we extend and apply the pathwise sensitivity analysis method in [19] to biochemical reaction networks, and demonstrate the intrinsic sensitivity structure of the network. Such systems are typically modeled as jump Markov processes and they are simulated using either exact algorithms such as the Stochastic Simulation Algorithm (SSA), [20-22] and the next-reaction method [23], or by employing approximations such as mean field ODEs, tau-leap [24] and stochastic Langevin methods [25].

We show that the proposed pathwise method allows us to discover the intrinsic sensitivities of the reaction network by decomposing the FIM into diagonal blocks. The block-diagonal structure of the proposed FIM reveals, in a straightforward way, the sensitivity interdependencies between the system parameters. For instance, if each propensity function depends only on one parameter –usually the reaction constant– then the FIM is a diagonal matrix (see (14)). The sparse representation of the FIM can be essential in optimal experimental design as well as in parameter identifiability and robustness where each subset of the parameters defined by a block of the FIM can be treated separately. Moreover, our earlier rigorous analysis [19] for the stationary regime suggests suitable extensions in the transient case which are here tested and validated. Finally, we present strategies for efficiently and reliably implementing the proposed method for high-dimensional, complex stochastic systems using an array of existing accelerated versions of the SSA algorithm such as mean field, stochastic Langevin, *τ*-leap approximations and their variants, [21,24-27].

We test the proposed set of methods and computational strategies in three examples of biochemical networks. First, we consider a prototypical protein production/degradation model, i.e, a single-species birth/death model, with explicitly known formulas for the stationary and the time-dependent distribution. This model serves as a benchmark where the differences between the proposed pathwise FIM and the stationary FIM are highlighted. Second, we study the parameter sensitivities of a p53 gene model for cell cycle regulation and response to DNA damage, that incorporates the feedback between the tumor suppressor p53 gene and the oncogene Mdm2 [28]. This is a reaction network that exhibits random oscillations in its steady state, and for which continuum approximations of the SSA such as LNA break down due to persistent oscillations between high and low populations. Using the proposed method, we also study a far more complex network, the epidermal growth factor receptor (EGFR) model, describing signaling processes between mammalian cells [29-31]. This is a high-dimensional system both in the number of variables and parameters, including 94 species and 207 reactions. Having a gradient-free method for this example with parameter space of dimension 207 provides a significant advantage over gradient methods such as finite differencing, where the computation of a very high number of partial derivatives and/or directional derivatives is needed and with possibly significant variance that scales with the dimension, [11]. By contrast, the eigenvalue/eigenvector analysis of the proposed FIM identifies the order from least to most sensitive directions (determined by the eigenvectors of the FIM) by the corresponding eigenvalues.

In Methods, we present the derivation of the Relative Entropy Rate and its corresponding Fisher Information Matrix for continuous-time jump Markov processes as well as we reveal the block-diagonal structure of the FIM for commonly encountered reaction networks, continued by the presentation of both unbiased and biased –but accelerated– statistical estimators for RER and FIM. Then, in the Results, we apply and validate the proposed pathwise sensitivity analysis methodology in three complex biological reaction networks.

## Methods

We consider a well-mixed reaction network with *N* species, **S** = {*S*_1_, …, *S*_*N*_}, and *M* reactions, **R** = {*R*_1_, …, *R*_*M*_}. The state of the system at any time *t* ≥ 0 is denoted by an *N*-dimensional vector **X**(*t*) = [*X*_1_(*t*), …, *X*_*N*_(*t*)]^*T*^ where *X*_*i*_(*t*) is the number of molecules of species *S*_*i*_ at time *t*. Let the *N*-dimensional vector *ν*_**j**_ correspond to the stoichiometry vector of *j*-th reaction such that *ν*_*i*,*j*_ is the stoichiometric coefficient of species *S*_*i*_ in reaction *R*_*j*_. Given that the reaction network at time *t* is in state **X**(*t*) = **x**, the propensity function, *a*_*j*_(**x**), is defined so that the infinitesimal quantity *a*_*j*_(**x**)*dt* gives the transition probability of the *j*-th reaction to occur in the time interval [*t*, *t* + *dt*]. Propensities are typically dependent on the state of the system and the reaction conditions (i.e., external parameters) of the network such as temperature, pressure, etc. Mathematically, ${\left\{\text{X}(t)\right\}}_{t\in R_{+}}$ is a continuous-time, time-homogeneous, jump Markov process with countable state space $E\subset N^{N}$. The transition rates of the Markov process are the propensity functions *a*_*j*_(·), *j* = 1, …, *M*. The transition rates determine the clock of the updates (or jumps) from a current state **x** to a new (random) state **x**^′^ through the total rate $a_{0}(\text{x}):=\sum_{j=1}^{M}a_{j}(\text{x})$ while the transition probabilities of the process are determined by the ratio $\frac{a_{j}(\text{x})}{a_{0}(\text{x})}$. We refer to *Algorithm* 1 for the details of the stochastic simulation.

### 

#### Relative entropy

Assume that two probability distributions (or more generally probability measures)  and $\tilde{P}$ have corresponding probability densities *p* = *p*(*x*) and $\tilde{p}=\tilde{p}(x)$. Then, the Relative Entropy or Kullback-Leibler divergence of  with respect to $\tilde{P}$ is defined as [32,33]

(1) $$R\left(P|\tilde{P}\right):=\int p(x)\log\left(\frac{p(x)}{\tilde{p}(x)}\right)\text{dx}\;.$$

In a more general setting, relative entropy is defined as $R\left(P|\tilde{P}\right):=\int \log\left(\frac{dP}{d\tilde{P}}\right)\;dP$ where $\frac{dP}{d\tilde{P}}$ is a function known as Radon-Nikodym derivative while the integration is performed with respect to the probability measure , [34]. A necessary condition for the relative entropy to be well-defined is that the Radon-Nikodym derivative exists which is satisfied when  is absolutely continuous with respect to $\tilde{P}$. Relative entropy has been utilized in a diverse range of scientific fields from statistical mechanics [34] to coding in telecommunications (information theory) [33] and finance [35], and it possesses the following three fundamental properties: 

(i) it is always non-negative,

(ii) it equals to zero if and only if $P=\tilde{P}P$-almost everywhere, and,

(iii)

$$R\left(P|\tilde{P}\right)<\infty$$

if and only if  and $\tilde{P}$ are absolutely continuous with respect to each other.

From an information theory perspective, relative entropy quantifies the loss of information when $\tilde{P}$ is utilized instead of , [33]. In other words, relative entropy quantifies the inefficiency of assuming an incorrect or perturbed distribution $\tilde{P}$ instead of employing the true distribution . Therefore, even though not a metric, relative entropy has been used as a suitable quantity for the assessment of parametric sensitivities since the higher the relative entropy (i.e., the information loss) in some perturbed direction, the larger the sensitivity should be in this direction.

#### Pathwise relative entropy and relative entropy rate

Proceeding to the pathwise formulation of the relative entropy, we assume that the propensities depend on a parameter vector $\theta \in R^{K}$ (i.e., $a_{j}(\text{x})\equiv a_{j}^{\theta }(\text{x})$) while the continuous-time jump Markov process ${\left\{\text{X}(t)\right\}}_{t\in R_{+}}$ lies in the *stationary regime*. We denote by *μ*^*θ*^(**x**) the steady state (or stationary) distribution of the stochastic process **X**(*t*). The stationary path distribution of the process in the interval [0, *T*] is denoted by $Q_{\left[0,T\right]}^{\theta }$. Notice that path distributions (i.e., time-series distributions) are high-dimensional complex objects; for instance, if we consider the simpler discrete-time Markov chain, ${\left\{{\text{Z}}_{n}\right\}}_{n\in Z_{+}}$, defined by the transition probability density *p*(**z**, **z**^′^), then, utilizing repeatedly the Markov property, the stationary path distribution of the time-series (**z**_0_, **z**_1_, …, **z**_*T*_) is given by

$$\begin{array}{ll} \;Q_{\left[0,T\right]}({\left\{{\text{Z}}_{n}={\text{z}}_{n}\right\}}_{0\leq n\leq T}) & =\text{Prob}(z_{0},\ldots ,z_{T})\\ & =\mu ({\text{z}}_{0})p({\text{z}}_{0},{\text{z}}_{1})\ldots p({\text{z}}_{T-1},{\text{z}}_{T}). \end{array}$$

Proceeding, we consider another continuous-time jump Markov process ${\left\{\tilde{X}(t)\right\}}_{t\in R_{+}}$ defined by perturbing the propensity functions by a small vector $\varepsilon \in R^{K}$. The corresponding steady state and path distributions of ${\left\{\tilde{X}(t)\right\}}_{t\in R_{+}}$ are denoted by *μ*^*θ*+*ε*^(**x**) and $Q_{\left[0,T\right]}^{\theta +\varepsilon }$, respectively. Let the two path distributions $Q_{\left[0,T\right]}^{\theta }$ and $Q_{\left[0,T\right]}^{\theta +\varepsilon }$ be absolutely continuous with respect to each other which is satisfied when $a_{j}^{\theta }(\text{x})=0$ if and only if $a_{j}^{\theta +\varepsilon }(\text{x})=0$ holds for all **x** ∈ *E* and *j* = 1, …, *M*. Then, the Relative Entropy of the path distribution $Q_{\left[0,T\right]}^{\theta }$ with respect to $Q_{\left[0,T\right]}^{\theta +\varepsilon }$ is defined similarly to (1) as

(2) $$R\left(Q_{\left[0,T\right]}^{\theta }\;|\;Q_{\left[0,T\right]}^{\theta +\varepsilon }\right):=\int \log\left(\frac{dQ_{\left[0,T\right]}^{\theta }}{dQ_{\left[0,T\right]}^{\theta +\varepsilon }}\right)\;dQ_{\left[0,T\right]}^{\theta },$$

where $\frac{dQ_{\left[0,T\right]}^{\theta }}{dQ_{\left[0,T\right]}^{\theta +\varepsilon }}$ is the Radon-Nikodym derivative of $Q_{\left[0,T\right]}^{\theta }$ with respect to $Q_{\left[0,T\right]}^{\theta +\varepsilon }$. In fact, using the Girsanov’s formula, we can obtain an explicit expression for the Radon-Nikodym derivative in terms of the propensities, [34]. In the context of sensitivity analysis, the pathwise relative entropy $R\left(Q_{\left[0,T\right]}^{\theta }\;|\;Q_{\left[0,T\right]}^{\theta +\varepsilon }\right)$ is a measure of information loss due to an *ε*-perturbation of the model parameters, and consequently it is a natural measure of parametric sensitivity.

Moreover, in the stationary regime, relative entropy increases linearly in time, hence the Relative Entropy Rate (RER) which is the time average of the relative entropy,

(3) $$H\left(Q^{\theta }\;|\;Q^{\theta +\varepsilon }\right):=\lim_{T\to \infty }\frac{1}{T}R\left(Q_{\left[0,T\right]}^{\theta }\;|\;Q_{\left[0,T\right]}^{\theta +\varepsilon }\right),$$

is a well-defined quantity, [36]. As first proposed in [19], $H\left(Q^{\theta }|Q^{\theta +\varepsilon }\right)$ is a suitable time-independent measure of sensitivity: it measures the rate of the loss of information due to an *ε*-perturbation of the model parameters, in the long-time, stationary dynamics regime of the stochastic process. Furthermore, RER admits an explicit formula given by (see Additional file 1 for a rigorous derivation)

(4) $$\begin{array}{l} H\left(Q^{\theta }|Q^{\theta +\varepsilon }\right)=E_{\mu^{\theta }}\left[\overset{M}{\underset{j=1}{\sum }}a_{j}^{\theta }(\text{x})\log\frac{\overset{\theta }{\underset{j}{a}}(\text{x})}{a_{j}^{\theta +\varepsilon }(\text{x})}\right)\\ \;-\left(\left(a_{0}^{\theta }(\text{x})-a_{0}^{\theta +\varepsilon }(\text{x})\right)\right]. \end{array}$$

Thus, from a practical point of view, RER is an observable of the stochastic process which can be computed numerically as an ergodic average, requiring only the knowledge of the propensity functions and the stoichiometric matrix (*ν*)_*i*,*j*_. Nevertheless, in order to carry out the sensitivity analysis in the parameter vector *θ*, the computation of RER for different *ε*’s is necessary which can be computationally challenging for high-dimensional parameter spaces. Thus, a sensitivity analysis methodology which does not depend on *ε*’s –such methods are called “gradient-free”– is desirable and is developed next.

#### Pathwise Fisher information matrix

Even though not directly evident from (4), a Taylor series expansion of RER in terms of *ε* reveals that RER is locally a quadratic function of the parameter vector $\varepsilon \in R^{K}$. Indeed, RER is non-negative when *ε* ≠ 0 and equals to zero when *ε* = 0 thus the linear term in the Taylor expansion is zero. Therefore, RER is written –under smoothness assumptions on the propensity functions with respect to the parameter vector *θ*– as [19],

(5) $$H\left(Q^{\theta }|Q^{\theta +\varepsilon }\right)=\frac{1}{2}\varepsilon^{T}F_{H}(Q^{\theta })\varepsilon +O(|\varepsilon {|}^{3}),$$

where $F_{H}(Q^{\theta })$ is a *K* × *K* matrix that can be considered as a pathwise analogue for the steady state Fisher Information Matrix (FIM). Similarly to the steady state FIM for parametrized distributions [33], $F_{H}(Q^{\theta })$ is the Hessian of the RER which geometrically corresponds to the curvature around the minimum value of the relative entropy rate. The pathwise FIM contains up to third order accuracy all the sensitivity information for the path distribution at point *θ* for any perturbation direction *ε*, therefore, the computation of the FIM is sufficient up to third order for the evaluation of all the local sensitivities of the path distribution around the parameter vector *θ*. Moreover, an explicit formula for the pathwise FIM is given by (see Additional file 1 for a derivation)

(6) $$\;F_{H}(Q^{\theta }):=E_{\mu^{\theta }}\left[\overset{M}{\underset{j=1}{\sum }}a_{j}^{\theta }(\text{x})\nabla_{\theta }\log\overset{\theta }{\underset{j}{a}}(\text{x})\nabla_{\theta }\log a_{j}^{\theta }{\left(\text{x}\right)}^{T}\right].$$

The implications of this explicit formula are twofold. First, it reveals that for many typical reaction networks the FIM has a special block-diagonal structure which reflects the parameter interdependencies and it is discussed in detail below. Second, the FIM is based on the propensity functions as well as on their derivatives which are known – actually, they define the process– thus the FIM, similarly to RER, is numerically computable as an observable of the process. Subsequent sections present various strategies to numerically estimate both the RER and the FIM in an efficient fashion.

Furthermore, the pathwise FIM, $F_{H}(Q^{\theta })$, can be used not only for the estimation/approximation of RER via (5) but also to infer intrinsic knowledge for the system’s sensitivities [16,37]. In general, the spectral analysis of the FIM reveals the (local) *comparatively* most/least sensitive directions of the system around *θ*. Indeed, by ordering the eigenvalues of the FIM as

$$\lambda_{1}^{\theta }\geq \ldots \geq \lambda_{K}^{\theta }\geq 0,$$

it can be inferred that the most sensitive direction corresponds to the eigenvector with eigenvalue $\lambda_{1}^{\theta }$ while the least sensitive direction corresponds to the eigenvector with eigenvalue $\lambda_{K}^{\theta }$. Additionally, the FIM is one of the most useful tools for optimal experimental design. Many of the optimality criteria such as D-optimality where the determinant of the FIM is maximized or A-optimality where the trace of the inverse of the FIM is minimized are based on FIM, [37].

In the same direction, robustness of the system to parameter perturbations or errors as well as parameter identifiability can be studied utilizing spectral analysis of the FIM. For instance, parameter identifiability is satisfied when all the eigenvalues of the FIM are above a given threshold, [18].

#### Sensitivity analysis at the logarithmic scale

In many biochemical reaction networks, the model parameters differ by orders of magnitude and a reasonable option for carrying out sensitivity analysis is to perform perturbations which are proportional to the parameter magnitude. This can be carried out by perturbing the logarithm of the model parameters instead of the parameters itself. Using the chain rule ∇_log*θ*_*f*(*θ*) = ∇_*θ*_*f*(*θ*).∇_log*θ*_*θ* = *θ*.∇_*θ*_*f*(*θ*) where ‘. ’ is defined as the element by element multiplication (i.e., (*a*.*b*)_*k*_ = *a*_*k*_*b*_*k*_, *k* = 1, …, *K*), we obtain the logarithmically-scaled FIM:

(7) $${\left(F_{H}(Q^{\log\theta })\right)}_{k,l}=\theta_{k}\theta_{l}{\left(F_{H}(Q^{\theta })\right)}_{k,l}\;,\;k,l=1,\ldots ,K\;,$$

where $F_{H}(Q^{\theta })$ is given by (6). Similarly, the logarithmic perturbation for the RER is carried out using the perturbation vector *θ*.*ε* instead of *ε*. Notice that (5) continues to be valid for the logarithmic scale, i.e.,

(8) $$H\left(Q^{\theta }|Q^{\theta +\theta .\varepsilon }\right)=\frac{1}{2}\varepsilon^{T}F_{H}(Q^{\log\theta })\varepsilon +O(|\varepsilon {|}^{3})\;.$$

#### Linking relative entropy and observables

As we discussed in the previous sections, relative entropy provides a mathematically elegant and computationally tractable methodology for the parameter sensitivity analysis of complex, stochastic dynamical systems. Such results focus on the sensitivity of the entire probability distribution, either at equilibrium or at the path-space level, i.e., for the entire stationary time-series. However, in many situations of chemical and biological networks, the interest is focused on observables such as mean populations, population correlations, population variance as well as path-space observables such as time autocorrelations and extinction times. Therefore, it is plausible to attempt to connect the parameter sensitivities of observables to the relative entropy methods proposed here. Indeed, relative entropy can provide an upper bound for a large family of observable functions through the Pinsker (or Csiszar-Kullback-Pinsker) inequality, [33].

More precisely, for any bounded observable function *f*, the Pinsker inequality states that

(9) $$|E_{Q^{\theta }}[f]-\;E_{Q^{\theta +\varepsilon }}[f]|\leq ||f|{|}_{\infty }\sqrt{2R\left(Q^{\theta }|Q^{\theta +\varepsilon }\right)},$$

where ||·||_*∞*_ denotes the supremum (here, maximum) of *f*. An obvious outcome of this inequality is that if the (pseudo-)distance between two distributions defined by $R\left(Q^{\theta }|Q^{\theta +\varepsilon }\right)$ is controlled, then the error between the two distributions is also controlled for any bounded observable. In the context of sensitivity analysis, inequality (9) states that if the relative entropy is small, i.e., insensitive in a particular parameter direction, then, any bounded observable *f* is also expected to be insensitive towards the same direction. In this sense, (9) can be viewed as a “conservative” –but not necessarily sharp– bound for the parametric sensitivity analysis of observables, including path-dependent observables such as long-time averages and autocorrelations.

From a practical perspective, (9) can be used as an indicator that suggests –even in the presence of a very high-dimensional parameter space– which are the insensitive parameter directions for observables of stochastic dynamical systems. The least-sensitive directions can be verified computationally and we present in the sequel two examples of this practical strategy in the p53 and the EGFR models. More generally, determining insensitive directions in parameter space can by particularly useful in multi-parameter models in systems biology characterized by “sloppiness”, i.e., when most model parameters allow for a vast range of perturbations without affecting the dynamics, [38]. As we concretely show in the EGFR example, our methodology can easily demonstrate and quantify such properties in stochastic dynamics through the use of the spectral analysis of the pathwise FIM, even if the models include a very large number of parameters.

##### Remark 1

We note that in order to carry out such an analysis in a mathematically rigorous manner which includes both sensitive and insensitive parameters, we need to require that the norm ||·||_*∞*_ in (9) is controlled. For instance, typical observables in biochemical reaction networks are the number of molecules for each species, hence *f*(*x*) = *x*. Thus, for reaction networks where the population size is large, the Pinsker inequality (9) will provide a bound that may not be sharp. In fact, it was recently shown that there are alternative bounds which are expected to be practically more useful than (9) in the sense that they provide sharp upper bounds for observables in terms of the relative entropy, [39]. These sharp bounds rely on the variational representation of the relative entropy and the existence of an explicit minimizer for the upper bound. Furthermore, [39] combined with our work on RER and pathwise FIM suggests new mathematical questions towards pursuing practical sharp upper bounds involving RER and pathwise FIM. In view of these comments, we conclude that (9), (a) constitutes a theoretical indicator that relative entropy is a reliable tool for sensitivity analysis and more generally for uncertainty quantification; (b) from a practical perspective, it is capable to rule out insensitive directions in parameter space, which in turn provides a significant advantage in the study of “sloppy” multi-parameter models.

### Block-diagonal structure of the pathwise FIM

**Figure 1:**
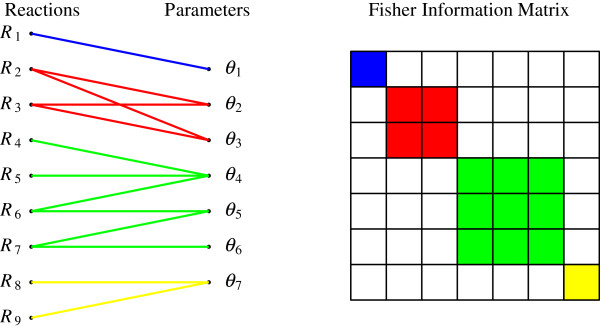
**The graph representation (left panel) of the dependencies between the reactions (left column) and the model parameters (right column) as well as the corresponding block-diagonal structure of the FIM (right panel).** The grouping of the parameters is carried out by noting the connected parts of the graph. In this example *K* = 7 while the largest dimension of the blocks is *L* = 3.

In chemical reaction networks, reactions typically depend only on a small subset of the parameter vector. Mathematically, this is described as

(10) $$a_{j}^{\theta }(\text{x})=a_{j}(\text{x};\theta_{k_{1}},\ldots \theta_{k_{L_{j}}}),$$

where $k_{1},\ldots ,k_{L_{j}}\in \{1,\ldots ,K\}$ while *L*_*j*_ ≪ *K* is the number of involved parameters in reaction *R*_*j*_. Using (6), it can be shown that this parametric dependence of the propensities is directly reflected on the pathwise FIM. Indeed, after grouping the reactions into subsets in such a way that each subset contains the minimum number of reactions having common parameters, the pathwise FIM – upon rearrangement of the parameter vector– becomes a block-diagonal matrix. The pathwise FIM is then written as

(11) $$F_{H}(Q^{\log\theta })=\left[\begin{array}{ll} A_{1}^{\theta } & \text{0}\\ \;\ddots \\ \text{0} & A_{I}^{\theta } \end{array}\right]$$

where $A_{1}^{\theta },\ldots ,A_{I}^{\theta }$ are block matrices. The block matrices which are defined by the reaction subsets with the same parametric dependence are easily obtained by creating a graph whose nodes are the reactions and the parameters while the edges are their dependences. Then, the parameter nodes contained in a connected subgraph define a parameter subset which in turn corresponds to a block of the FIM. An illustration of this procedure is shown in Figure 1 where a reaction network with *M* = 9 reactions and *K* = 7 parameters is plotted. The parametric dependencies of the reactions are shown in the left panel where 4 subgroups of parameters are defined based on the graph connectivity. The resulting block-diagonal structure of the FIM is shown on the right panel of Figure 1.

Before proceeding with the theoretical computation of the FIM for various well-known classes of biochemical reaction networks, we list some of the implications of this simplified structure of the FIM in sensitivity analysis and elsewhere. 

(i) The sparsity of the FIM is proportional to the parametric decoupling between the reactions. Knowing a priori the zero elements of the FIM, there is no need to numerically compute them. It is clear that the computation cost for each sample drops from *O*(*K*^2^) to *O*(*KL*) where *L* is the largest dimension of the block matrices.

(ii) The inverse of the FIM is also block-diagonal and each block of the inverse FIM is the inverse of the respective block. This fact allows us in the parameter estimation problem to easily evaluate the lower bound of the variance, at least for the complete-data case [40], i.e., obtain Cramer-Rao bounds, [41,42] which are given by the diagonal elements of the inverse of the FIM.

(iii) Relation (11) implies that that optimality criteria in optimal experiment design, [37], are significantly simplified. For example, the determinant employed in the D-optimality test is given by the relation $\det(F_{H})=\prod_{i=1}^{I}\det(A_{i})$, while the trace of the inverse of the FIM utilized in the A-optimality reduces to $\text{tr}({F_{H}}^{-1})=\sum_{i=1}^{I}\text{tr}(A_{i}^{-1})$.

(iv) Given that parametric identifiability is characterized by the magnitude of the eigenvalues of the FIM, e.g. a zero eigenvalue corresponds to a non-identifiable direction in parameter space, [18,43], then the block-diagonal structure (11) can provide additional insights in parameter identification. For instance, the identifiability of the parameters of the group that corresponds to the *i*-th block, *A*_*i*_, will be increased if the smaller eigenvalues of the *i*-th block can be increased. On the other hand, if the determinant of the *i*-th block equals to zero then at least one of its eigenvalues is zero and thus the corresponding linear combinations of parameters are non-identifiable. Similarly, the robustness of the system to perturbations of the parameters of the *i*-th group will be increased if there is a way to decrease the larger eigenvalues of the *i*-th block.

Overall, we note that extracting useful information regarding model parameters can be performed for each block of the pathwise FIM independently. Next, we discuss two specific examples of biochemical reaction networks where the explicit calculation of the block-diagonal FIM is demonstrated.

#### Reactions with independent reaction constants (mass action kinetics)

An important class of well-mixed reaction networks take the general form “$\alpha_{j}A_{j}+\beta_{j}B_{j}\overset{\theta_{j}}{\to }\ldots$” where *A*_*j*_ and *B*_*j*_ are the reactant species while *α*_*j*_ and *β*_*j*_ are the respective number of molecules needed for the reaction. The reaction constant, *θ*_*j*_, is the parameter of the *j*-th reaction. The propensity function for the *j*-th reaction is given as the product between a rate constant and a function of the current state **x**:

(12) $$a_{j}(\text{x})=\theta_{j}g_{j}(\text{x}),\;j=1,\ldots ,M\;.$$

Typically, gj(x)=xAjαjxBjβj which stems from the law of mass action, however, it can take different forms depending on the modeling of the physical process. This reaction network has *K* = *M* parameters, while each propensity depends only on one parameter, i.e., *L*_*j*_ = 1 in (10) for *j* = 1, …, *M*. The (*k*, *l*)-th element of the FIM in the logarithmic scale is explicitlyl given by

(13) $$\;\begin{array}{l} {\left(\;F_{H}(Q^{\log\theta })\;\right)}_{k,l}=\theta_{k}\theta_{l}\\ \;\times E_{\mu^{\theta }}\left[\overset{M}{\underset{j=1}{\sum }}a_{j}^{\theta }(\text{x})\partial_{\theta_{k}}\;\log a_{j}^{\theta }(\text{x})\partial_{\theta_{l}}\log a_{j}^{\theta }{\left(\text{x}\right)}^{T}\;\right]\;, \end{array}$$

where *μ*^*θ*^ is the stationary distribution of the stochastic process. Furthermore, it holds that $\partial_{\theta_{k}}\log a_{j}^{\theta }(\text{x})=\frac{1}{\theta_{k}}\delta_{k}(j)$ where *δ*(·) is the Dirac function, therefore the pathwise FIM is a diagonal matrix with elements given by

(14) $${\left(F_{H}(Q^{\log\theta })\right)}_{k,l}=\left\{\begin{array}{ll} E_{\mu^{\theta }}\left[a_{k}^{\theta }(\text{x})\right]\;, & l=k\\ \;0\;, & l\neq k \end{array}\;.\right)$$

This result demonstrates that the sensitivity of a reaction constant is proportional to the equilibrium average of the respective propensity function. Moreover, due to the diagonal form of the FIM, it is straightforward to carry out the eigenvalue analysis and infer the most/least sensitive directions of the reaction network: the eigenvalues of the FIM are its diagonal elements while the eigenvectors are the standard basis vectors of $R^{K}$. Hence, the most (respectively least) sensitive parameter is obtained from the largest (respectively smallest) diagonal element of the FIM. Furthermore, (14) demonstrates that the (local) robustness of the reaction network to a specific parameter is inversely proportional to the mean propensity of the corresponding reaction. Another observation stemming from the diagonal structure of the pathwise FIM is that each rate constant can be estimated from time-series data independently from the other rate constants. This observation has been already pointed out and discussed in the context of maximum likelihood estimation for the complete-data case ([40], Sec. 10.2).

Additionally, the mean firing rate of a reaction is equal to the mean propensity. Hence, it can be stated that the parameters that correspond to the faster reactions, i.e., to reactions with larger mean firing rate, are more sensitive in a pathwise entropy sense. It should be noted, however, that not all observables are sensitive to the parameters that correspond to the faster reactions and there are examples (see the protein production-degradation model in the Results section) where steady state observables such as the equilibrium distribution remain insensitive to specific perturbation directions even though their mean propensity may be increased. Finally, we would like to remark that even though $E_{\mu^{\theta }}\left[a_{k}^{\theta }(\text{x})\right]=\theta_{k}E_{\mu^{\theta }}\left[g_{k}^{\theta }(\text{x})\right]$ trivially holds true, the diagonal elements of the FIM are not linear functions of the corresponding reaction constants since the steady state distribution *μ*^*θ*^, depends also on the parameter vector *θ*. In fact, high reaction constants do not necessarily imply large mean propensities and hence a more sensitive parametric dependence. This is specifically due to the mean value in (14) and as an illustrative example we refer to the simple protein production-degradation model (e.g., compare (24) and (27)).

#### Michaelis-Menten kinetics

Another class of reaction networks is the Michaelis-Menten kinetics. In its simplest form (e.g., single-substrate reaction without intermediate), this chemical network contains a reaction between species *A* and *B* (i.e., *A* → *B*) with propensity function given by

$$a_{k}^{\theta }(\text{x})=\frac{\theta_{k}{\text{x}}_{A}}{\theta_{k^{\prime }}+{\text{x}}_{A}}.$$

This reaction sub-network which is derived under a quasi-steady-state assumption is one of the best-known models of enzyme kinetics in biochemistry [44]. The parameter *θ*_*k*_ (usually denoted by *V*_max_) represents the maximum rate achieved by the system, at maximum (saturating) substrate concentrations while the Michaelis constant $\theta_{k^{\prime }}$ (usually denoted by *K*_*m*_) is the substrate concentration at which the reaction rate is half the maximum value. The propensities of this Michaelis-Menten sub-network depend on two parameters (*L*_*k*_ = 2 in (10)) thus the corresponding FIM block is a 2 × 2 matrix. The elements of the FIM matrix are given by

(15) $${\left(F_{H}(Q^{\log\theta })\right)}_{k,l}=\left\{\begin{array}{cc} E_{\mu^{\theta }}\left[a_{k}^{\theta }(\text{x})\right]\;, & l=k\\ -\theta_{k^{\prime }}E_{\mu^{\theta }}\left[\frac{a_{k}^{\theta }(\text{x})}{\theta_{k^{\prime }}+{\text{x}}_{A}}\right]\;, & l=k^{\prime }\\ \;0\;, & l\neq k,k^{\prime } \end{array}\right)$$

for the *k*-th row while the *k*^′^-th row is given by

(16) $${\left(F_{H}(Q^{\log\theta })\right)}_{k^{\prime },l}=\left\{\begin{array}{cc} \theta_{k^{\prime }}^{2}E_{\mu^{\theta }}\left[\frac{a_{k}^{\theta }(\text{x})}{{\left(\theta_{k^{\prime }}+{\text{x}}_{A}\right)}^{2}}\right]\;, & l=k^{\prime }\\ -\theta_{k^{\prime }}E_{\mu^{\theta }}\left[\frac{a_{k}^{\theta }(\text{x})}{\theta_{k^{\prime }}+{\text{x}}_{A}}\right]\;, & l=k\\ \;0\;, & l\neq k^{\prime },k\;\;. \end{array}\right)$$

In general, biochemical reaction networks may have significantly more complex propensities, nevertheless, the computation of the FIM follows exactly the same calculation lines for any propensity function.

### Strategies for the statistical estimation of RER and FIM

Previous sections introduced and justified RER and FIM as appropriate observables for measuring the sensitivity analysis of the reaction network’s parameters in long-time dynamics. This section presents strategies on how to efficiently estimate these quantities as ergodic averages of the underlying stochastic process.

#### Unbiased statistical estimators

Since the stationary distribution, *μ*^*θ*^, is usually not known, both FIM and RER should be estimated numerically as ergodic averages. Indeed, the statistical ergodic estimator for RER is given by

(17) $$\begin{array}{l} {\bar{H}}^{\left(n\right)}=\frac{1}{T}\overset{n-1}{\underset{i=0}{\sum }}\Delta t_{i}\left[\overset{M}{\underset{j=1}{\sum }}a_{j}^{\theta }({\text{x}}_{i})\log\frac{a_{j}^{\theta }({\text{x}}_{i})}{a_{j}^{\theta +\varepsilon }({\text{x}}_{i})}\right)\\ \;-\left(\left(a_{0}^{\theta }({\text{x}}_{i})-a_{0}^{\theta +\varepsilon }({\text{x}}_{i})\right)\right] \end{array}$$

where *Δt*_*i*_ is an exponential random variable with parameter given by the total rate, $a_{0}^{\theta }({\text{x}}_{i})$, while $T=\overset{n}{\underset{i=1}{\sum }}\Delta t_{i}$ is the total simulation time. The sequence ${\left\{{\text{x}}_{i}\right\}}_{i=0}^{n}$ is the embedded Markov chain with transition probabilities from state **x**_*i*_ to state **x**_*i*+1_ is given by the ratio $\frac{a_{j}^{\theta }({\text{x}}_{i})}{a_{0}^{\theta }({\text{x}}_{i})}$. The weight *Δt*_*i*_, which is the waiting time at state **x**_*i*_, is necessary for the unbiased estimation of the observable, [45]. Similarly, the unbiased estimator for the FIM is

(18) $$\;\;{\bar{F}}_{H}^{\left(n\right)}=\frac{1}{T}\overset{n-1}{\underset{i=0}{\sum }}\Delta t_{i}\overset{M}{\underset{j=1}{\sum }}a_{j}^{\theta }({\text{x}}_{i})\nabla_{\theta }\log a_{j}^{\theta }({\text{x}}_{i})\nabla_{\theta }\log a_{j}^{\theta }{\left({\text{x}}_{i}\right)}^{T}.$$

Noticing that the computation of the local propensity functions $a_{j}^{\theta }({\text{x}}_{i})$ for all *j* = 1, …, *M* is needed for the simulation of the jump Markov process when Monte Carlo methods such as SSA [45] is utilized, the computation of the perturbed transition rates is the only additional computational cost for the numerical RER while the additional cost for the estimation of the FIM is the computation of the derivatives of the propensities. *Algorithm *1 summarizes the numerical computation of RER and FIM, employing the SSA for the simulation of the jump Markov process.

#### Accelerated statistical estimators

A typical feature of biochemical systems is that the modeled reaction network is large with hundreds or thousands of reactions and different time scales stemming from the orders of magnitude difference between the reaction rates and/or between the species concentrations, making the SSA extremely slow. A large number of multi-scale approximations of the original SSA have been developed in order to handle such issues resulting to accelerated simulation algorithms. For example, mean-field approximation ignores the fluctuations of the stochastic process and yields a deterministic system of ordinary differential equations (ODE) for the mean population of the species [46,47]. Stochastic corrections to the mean-field model such as stochastic Langevin [25] and linear noise approximation [48] can be applied in order to improve the accuracy of the simulation. An alternative approximation of the jump Markov process is the tau-leap method proposed by Gillespie [24] where a batch of events occurs at each time-increment, *τ*. Several improvements of the basic tau-leap algorithm on how to select adaptively the *τ*[49] or avoiding negative populations [27,50] have been proposed, however, their performance is heavily model-dependent.

##### **Algorithm 1** SSA-based numerical computation of RER and FIM

In this subsection, we propose such approximations in order to efficiently compute the FIM and/or RER observables, while maintaining controlled bias in the statistical estimators. As an illustration, we present the well-known mean-field approximation. The popularity of the mean-field modeling stems from their computational efficiency. To proceed, the stochastic process can be written as

(19) X(t)=x(t)+ηξ(t)

where *x*(*t*) is the deterministic part (mean) of the process, *ξ*(*t*) is the stochastic zero-mean part while *η* is the amplitude of the stochastic term. The amplitude of the stochastic term is proportional to the inverse square root of the reactant populations [25,48,51]. Thus, for large populations, the fluctuations of the time-evolving species populations become vanishingly small compared to the deterministic contributions. Consequently, the dominant part of the process is the deterministic term whose dynamics are governed by the ODE system

(20) $${\dot{x}}_{i}(t)=\overset{M}{\underset{j=1}{\sum }}\nu_{j,i}a_{j}^{\theta }(x(t))\;,\;i=1,\ldots ,N\;.$$

This ODE system is also known as reaction rate equations [25]. Restricted for simplicity to the special case with independent rate constants for each reaction, the diagonal elements of the FIM are approximated using (19) as

(21) $$\begin{array}{ll} \;{\left(F_{H}(Q^{\log\theta })\right)}_{k,k} & =E_{\mu^{\theta }}\left[a_{k}^{\theta }(\text{x})\right]\approx \frac{1}{T}\overset{n}{\underset{i=1}{\sum }}\Delta t_{i}a_{k}^{\theta }(\text{X}(t_{i}))\\ & =\frac{1}{T}\overset{n}{\underset{i=1}{\sum }}\Delta t_{i}\;a_{k}^{\theta }\left(x(t_{i})+\eta \xi (t_{i})\right)\\ & =\frac{1}{T}\overset{n}{\underset{i=1}{\sum }}\Delta t_{i}\;a_{k}^{\theta }(x(t_{i}))+O(\eta )\; \end{array}$$

Typically, such ODE system is solved using an adaptive time-step numerical integrator up to final time $T=\sum_{i=0}^{n}\Delta t_{i}$. Thus, for large species populations (|*S*_*i*_|≫1), the following numerical estimator for the FIM’s diagonal elements is obtained:

(22) $${\left({\bar{\bar{\text{F}}}}_{H}^{\left(n\right)}\right)}_{k,k}=\frac{1}{T}\overset{n}{\underset{i=1}{\sum }}\Delta t_{i}\;a_{k}^{\theta }(x(t_{i}))\;,\;k=1,\ldots ,K$$

Relation (22) suggests an algorithm similar to *Algorithm *1 for the numerical computation of the FIM where instead of SSA, an ODE solver is employed.

##### Remark 2

Multi-scale approximations are usually valid for large populations and relatively simple systems which do not exhibit complex dynamics such as bistability or intermittency. Indeed, large deviation arguments [52] or even explicitly available formulas for escape times [53] demonstrate that stochastic approximations cannot always capture correctly exit times, rare events, strong intermittency, etc. even in relatively simple systems. However, in order to simulate large biochemical systems there is often no other alternative than such approximate models, which nevertheless need to be employed with the necessary caution.

##### Remark 3

In biochemical systems, we are interested not only in the steady state, i.e., the stationary distribution or time-series, but also in the transient regime, e.g. signaling phenomena. The extension of the proposed sensitivity analysis method to the transient regime is justified by the fact that the time-normalized relative entropy can be also decomposed as a sum of simple integrals [33] which results to the fact that the statistical estimators (17) and (18) remain valid. In a subsequent section we present an example of a biochemical system (EGFR) which exhibits transient behavior, and where the proposed sensitivity analysis tools are tested and validated. The rigorous mathematical derivation of the relative entropy rate for the transient regime is out of the scope of this publication and a dedicated mathematical article on the time-dependent relative entropy rate will follow.

## Results and discussion

### A simple protein production/degradation model

We first consider an elementary stochastic model for protein production and degradation, [54], which is also a component of more complex models for gene regulatory networks, [55]. In this simplified model, the protein is produced at a constant rate *k*_1_, while it is degraded with rate *k*_2_, corresponding to the reactions

(23) $$\emptyset \underset{k_{2}}{\overset{k_{1}}{⇄}}X.$$

Accordingly, the corresponding propensity functions for the current state **x** = *x* are:

(24) $$a_{1}(x)=k_{1}\;\text{and}\;a_{2}(x)=k_{2}x\;.$$

We consider this simple stochastic model due to the available analytic representations of the steady state (equilibrium) distribution, time-dependent moments and autocorrelations, ([46], Sec. 7.1). Consequently, we can both illustrate the proposed pathwise sensitivity analysis, as well as compare it to the standard equilibrium FIM, revealing concretely differences between the two approaches.

The equilibrium distribution, *μ*^*θ*^, of this simple network is a Poisson distribution with parameter $\frac{k_{1}}{k_{2}}$. Therefore, the equilibrium FIM for the parameter vector *θ* = [*k*_1_, *k*_2_]^*T*^ is given in logarithmic scale by

(25) $$F_{R}(\mu^{\log\theta })=\frac{k_{1}}{k_{2}}\left[\begin{array}{ll} 1 & -1\\ -1 & 1 \end{array}\right].$$

On the other hand, the pathwise FIM is computed via (14):

(26) $$F_{H}(Q^{\log\theta })=k_{1}\left[\begin{array}{ll} 1 & 0\\ 0 & 1 \end{array}\right]\;,$$

where we used that

(27) $$E_{\mu^{\theta }}[\;a_{1}(x)]=E_{\mu^{\theta }}[\;a_{2}(x)]=k_{1}\;.$$

The complete calculations can be found in the Additional file 2. Some of the implications of the differences between these two FIMs are discussed next.

First, we observe that the equilibrium FIM, (25), is singular, i.e., one of the eigenvalues is zero. We readily see that in the parameter direction defined by the corresponding eigenvector, i.e., when the parameter ratio, $\frac{k_{1}}{k_{2}}$, remains constant, the system is expected to be insensitive, at least with respect to the equilibrium distribution. Clearly, this is a fact verified directly from the Poisson equilibrium distribution *μ*^*θ*^ which depends only on the ratio. On the other hand, the pathwise FIM, (26), is not singular and all the directions are equally sensitive. This fact suggests that observables for dynamic quantities may be sensitive not only to parameter ratio perturbations but also to other parameter perturbations. Indeed, one such example is the stationary autocorrelation function, which in the case of the simple protein production/degradation model is explicitly given by ([46], Sec. 7.1),

(28) $${<X_{t},X_{0}>}_{s}=\frac{k_{1}}{k_{2}}e^{-k_{2}t},$$

where < ·, · >_*s*_ denotes stationary averaging. Based on this formula, it is obvious that the autocorrelation function is also sensitive to *k*_2_, in addiction to the ratio $\frac{k_{1}}{k_{2}}$. This example demonstrates that in contrast to the pathwise FIM, the equilibrium FIM is inadequate to fully capture the dynamic properties of the process. Moreover, the pathwise FIM depends linearly only on *k*_1_, which shows that the reaction rate constants and propensity functions in (24) alone, can be misleading in the assessment of parametric sensitivity. Contrary, the mathematically correct equilibrium averaging of the propensities, i.e., (14) can lead to a completely different outcome, as can be readily seen when we compare (24) and (27).

In terms of parameter identifiability, the fact that one of the eigenvalues of (25) is zero implies that that the two-dimensional parameter vector of the system is non-identifiable. Indeed, the asymptotic normality of the maximum likelihood estimators, [41,42], states that their variance (also a lower bound according to the Cramer Rao theorem), which determines parameter identifiability of *k*_1_ and *k*_2_, is the reciprocal of the eigenvalues of (25). A straightforward calculation involving the eigenvectors of (25) shows that the only identifiable parameter is the ratio of the reaction constants appearing in (25). Therefore parametric inference for both parameters from equilibrium data is not possible. On the other hand, the pathwise FIM (26) is not singular, which readily implies that both parameters can be identified through (complete) time-series data, provided that *k*_1_ ≠ 0. Summarizing, this birth/death model is an example where equilibrium sampling is not enough for the identifiability of all the parameters, however, if dynamics data are available and are taken into account then all the parameters become identifiable as pathwise FIM asserts.

### The p53 gene model

**Figure 2:**
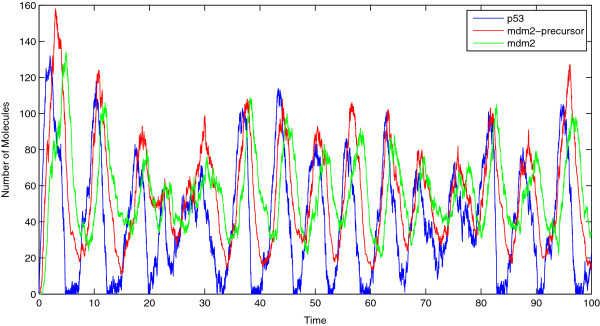
**Molecule concentration of p53, Mdm2-precursor and Mdm2.** Concentration oscillations as well as time delays (phase shifts) between the species are present due to the negative feedback loop. Furthermore, the concentration of p53 periodically approaches zero and since negative concentrations are not allowed, the stochastic characteristics of p53 are far from Gaussian.

The p53 gene plays a crucial role for effective tumor suppression in humans as its universal inactivation in cancer cells suggests [28,56,57]. The p53 gene is activated in response to DNA damage and gives rise to a negative feedback loop with the oncogene protein Mdm2. Models of negative feedback are capable of oscillatory behavior with a phase shift between the gene concentrations. Here, we perform sensitivity analysis to a simplified reaction network between three species, p53, Mdm2-precursor and Mdm2 introduced in [28]. The model consists of five reactions and seven parameters provided in Table 1. The nonlinear feedback regulator of p53 through Mdm2 takes place in the second reaction while the remaining four reactions fall in the special class where each reaction depends on one parameter. Due to these mechanisms a nontrivial steady state regime exists and can be characterized by random oscillations, see for instance Figure 2. The proposed sensitivity methodology is directly applicable, and the corresponding pathwise FIM, see (13) and Figure 1, consists of 5 diagonal blocks with respective size 1 × 1, 3 × 3, 1 × 1, 1 × 1, 1 × 1. Furthermore, the sensitivity analysis of this model has been performed earlier in [18] based on a linear noise approximation. Here, we present a detailed comparison between the two sensitivity analysis methodologies, since the one proposed here does not involve any approximation of the stochastic network dynamics.

**Table 1:** **The reaction table with *x * corresponding to p53,** ***y***_**0 **_**to Mdm2-precursor while *y *** **corresponds to Mdm2**

**Event**	**Reaction**	**Rate**	**Rate’s derivative**
*R*_1_	∅ → *x*	*a*_1_(**x**) = *b*_*x*_	∇_*θ*_*a*_1_(**x**) = [1, 0, 0, 0, 0, 0, 0]^*T*^
*R*_2_	*x* → ∅	$a_{2}(\text{x})=a_{x}x+\frac{a_{k}y}{x+k}x$	∇_*θ*_*a*_2_(**x**) = [0, *x*, *xy* / (*x* + *k*),
			−*a*_*k*_*xy* / (*x* + *k*)^2^, 0, 0, 0]^*T*^
*R*_3_	*x* → *x* + *y*_0_	*a*_3_(**x**) = *b*_*y*_*x*	∇_*θ*_*a*_3_(**x**) = [0, 0, 0, 0, *x*, 0, 0]^*T*^
*R*_4_	*y*_0_ → *y*	*a*_4_(**x**) = *a*_0_*y*_0_	∇_*θ*_*a*_4_(**x**) = [0, 0, 0, 0, 0, *y*_0_, 0]^*T*^
*R*_5_	*y* → ∅	*a*_5_(**x**) = *a*_*y*_*y*	∇_*θ*_*a*_5_(**x**) = [0, 0, 0, 0, 0, 0, *y*]^*T*^

The state of the reaction model is defined as **x** = [*x*, *y*_0_, *y* ]^*T*^ while the parameter vector is defined as *θ* = [*b*_*x*_, *a*_*x*_, *a*_*k*_, *k*, *b*_*y*_, *a*_0_, *a*_*y*_ ]^*T*^.

Figure 2 shows the time-series of the species for the parameter values in Table 2. Evidently, oscillatory behavior is observed at this parameter regime, where persistent random oscillations occur, ranging between high and low populations. On the other hand, the frequency of the oscillations is less variable as it has been already reported both experimentally and numerically [28]. Another interesting observation is that the concentration of p53 species usually attains the lower bound of its admissible value (populations cannot be negative) which results in stochastic effects far away from Gaussianity, as can be readily seen also in Figure 2.

**Table 2:** Parameter values for the p53 model

**Parameter**	***b***_***x***_	***a***_***x***_	***a***_***k***_	***k***	***b***_***y***_	***a***_**0**_	***a***_***y***_
Value	90	0.002	1.7	0.01	1.1	0.8	0.8

**Figure 3:**
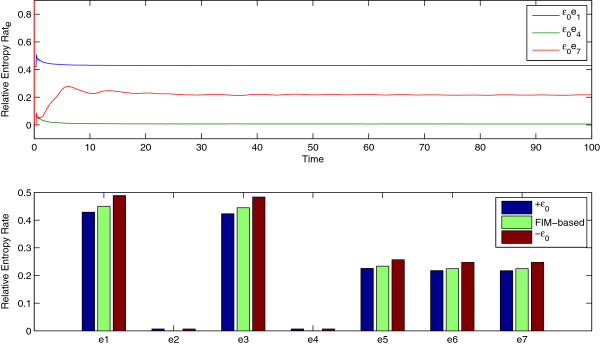
**RER in time (upper panel) for the parameter perturbation of *b***_***x***_** (blue), *k* (green) and *a***_***y***_** (red) by +10*%* (i.e.,*ε***_**0**_** = 0*.*1) as well as RER for various perturbation directions (lower panel) computed either directly (blue and red bars) or based on FIM (green bars).** Direction *e*_*k*_ corresponds to perturbation of parameter *θ*_*k*_.

Proceeding, we denote by *θ* = [*b*_*x*_, *a*_*x*_, *a*_*k*_, *k*, *b*_*y*_, *a*_0_, *a*_*y*_]^*T*^ the parameter vector. The numerical estimator for RER as well as for the pathwise FIM in the logarithmic scale are computed utilizing *Algorithm *1. Logarithmic sensitivity analysis is preferred because the range of the parameters values varies by orders of magnitude as can be seen in Table 2. The upper plot in Figure 3 shows the RER as a function of time for various perturbations. Viewing RER as an observable, it is striking the speed of relaxation of the estimator. Within two or three oscillation periods, RER has been converged to its value even though the three species have significant oscillations and stochasticity, as Figure 2 shows. A primary reason for the fast relaxation is the numerical estimator of RER where the summation is over all reactions even though only one reaction takes places at each jump (see (18)). Having the important property of fast convergence, global sensitivity analysis, where not only a point of the parameter regime but also large subsets of the parameter space, can be efficiently performed, [15]. The lower panel of Figure 3 shows the RER when only one of the parameters are perturbed by +10% or by -10%. Additionally, the RER computed from the FIM, utilizing (5), is also provided. The FIM approximation of RER is a second order approximation in terms of |*ε*|, hence the computation of FIM is typically enough to fully resolve the local sensitivities of a model. Evidently, the most sensitive parameters here are *b*_*x*_ and *a*_*k*_ while the least sensitive parameters are *a*_*x*_ and *k*.

#### Comparison to the LNA-based sensitivity approach

In [18], the authors suggested a linear noise approximation (LNA) for the stochastic evolution around the nonlinear mean-field equation, and based on this approximation a system of ODEs is derived for the mean and the covariance matrix of the approximation process. Since the noise of LNA is Gaussian, the mean and the covariance matrix contains all necessary information regarding the approximate stochastic model. Then, the associated FIM is derived and based on it, the sensitivities for each parameter are computed. Although there are regimes where this approximation is applicable (short times, high populations, systems with a single steady state, etc.), for systems with nontrivial long-time dynamics, e.g. metastable, it is not correct as large deviation arguments [52] and explicit formulas for escape times [53] show. Similar issues with non-gaussianity in the long-time dynamics arise in stochastic systems with strongly intermittent (pulse-like) or random oscillatory behavior [58]. In the p53 model considered in [18] which had the same parameter values as here, Figure 2 reveals that the time-series of the p53 populations persistently fluctuate between high and low values, thus the LNA approximation may not be accurate at least when the concentration of the species is very low.

**Figure 4:**
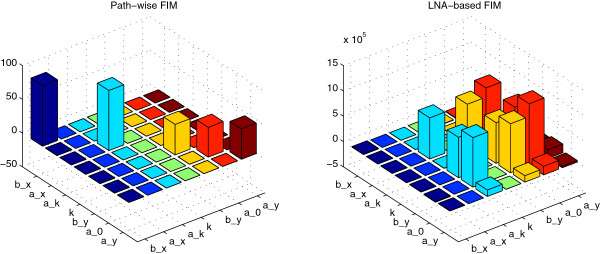
**The proposed pathwise FIM (left) based on RER as well as the (scaled) FIM based on LNA computed from the StochSens package **[59]**.** Evidently, the proposed method uncouples the parameter correlations since most of the off-diagonal elements are zero.

At first pass, when the parameters are grouped into two classes depending on their sensitivities, the two sensitivity approaches produce qualitatively similar results. Indeed, by visual inspecting the lower plot of Figure 3 in the current publication and Figure three in [18], the (more) sensitive parameters in both methods are *b*_*x*_, *b*_*y*_, *a*_*k*_, *a*_0_, *a*_*y*_ while the practically insensitive parameters are *a*_*x*_, *k*. However, upon closer inspection, the two methods produce different results. Figure 4 shows the proposed FIM (left) based on the exact (without any approximations) pathwise relative entropy theory, as well the FIM proposed in [18] which is derived from the LNA of the reaction system. The results are completely different and the proposed pathwise FIM is sparse as expected. A striking difference between the two sensitivity approaches is that the sensitivity of parameter *b*_*x*_ in our proposed method is relatively high compared to the other parameters while the sensitivity of *b*_*x*_ in the LNA-based method is at least one order lower compared to the other parameters, see Figure 4 (dark blue) and also compare Figure 3 of this publication and Figure three in [18].

**Figure 5:**
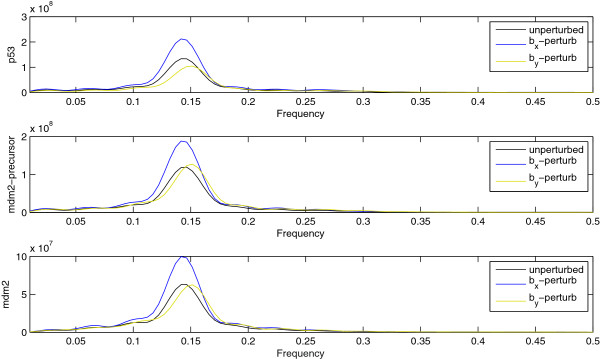
**Power Spectral Densities (PSD) of the time-series of the species in the p53 model for the unperturbed parameter regime (black), when *b***_***x ***_**is perturbed by +20% (blue) as well as when *b***_***y ***_**is perturbed by +20% (yellow).** The value and the position of the prominent peak of the PSD are related with the amplitude and the frequency of the species oscillations. The visual comparison between the averaged PSDs suggests that the spectral properties are more sensitive to *b*_*x*_ than to *b*_*y*_.

As a means of comparison between the methods, we perturb *b*_*x*_ as well as *b*_*y*_ by the same amount and observe the Power Spectral Density (PSD), i.e., the square of the absolute value of the Fourier transform of each species’ time-series. Given the sustained random oscillations observed in the p53 model, see Figure 2, the PSD is a suitable observable since it identifies the dominant periodicities and corresponding amplitudes in stationary time-series, [41]. Using the Pinsker inequality (9) as a guideline, we expect that the observable will not be sensitive to the least sensitive directions of the FIM, therefore, we focus on the most sensitive directions of the FIM identified in Figure 4. Figure 5 shows the averaged PSD for the three species of the model for the unperturbed case (black lines), the perturbation of *b*_*x*_ only (blue lines) as well as the perturbation of *b*_*y*_ only (yellow lines). One hundred realizations were used for the averaging procedure while the perturbation strength was +20%. The *L*_1_-norm, i.e., the integral of the absolute difference, between the unperturbed PSD and the *b*_*x*_-perturbation is 8.56 · 10^5^ while the *L*_1_-norm between the unperturbed PSD and and the *b*_*y*_-perturbation is 4.32 · 10^5^ which is about the half value. Hence, the averaged PSD –primarily the amplitude of the oscillations– is more sensitive to perturbations of *b*_*x*_ rather than to perturbations of *b*_*y*_ as our sensitivity analysis method predicted while the LNA-based method suggested the reverse order of sensitivity. We note that the choice of the *L*_1_ norm for the PSDs is justified since it describes the total energy (power) of the time-series, when viewed as a signal, [41]. An explanation of the performance of the LNA-based sensitivity analysis stems from the fact that the p53 species does not have Gaussian noise when the population is close to zero, and which can indeed occur frequently, see Figure 2 (blue line). Additionally, notice that both *b*_*x*_ and *b*_*y*_ affect the concentration of p53 explicitly or implicitly through the associated reactions thus their sensitivities are heavily biased due to the wrong statistical approximation of the p53 species. Moreover, we note that other observables, e.g., the arg max (location of the maximum) of the PSD, can be sensitive to perturbations of *b*_*y*_, see Figure 5. This observation is not contradictory to the findings of the proposed sensitivity methodology for two reasons: first, even though the Pinsker inequality (9) points towards the right direction regarding sensitive parameters for observables, it is only an upper (possibly crude) bound. Second, even though *b*_*x*_ is more sensitive in absolute value than *b*_*y*_ in terms of the proposed pathwise FIM, both *b*_*x*_ and *b*_*y*_ sensitivities have the same order of magnitude (see Figure 4) therefore there should exist observables which are also sensitive to *b*_*y*_ perturbations. Finally, for the sake of completeness, we report the observable values for the insensitive parameters. The *L*_1_-norm between the unperturbed PSD and the *a*_*x*_-perturbation is 9.03 · 10^3^ which is approximately two orders of magnitude less than the *L*_1_-norm for the sensitive parameters. Similar results hold for the other insensitive parameter, *k*. Thus, the outcome of the Pinkser inequality (9), i.e., that small RER values imply relatively insensitive parameters for the observables, is also numerically verified.

### Epidermal growth factor receptor model

**Figure 6:**
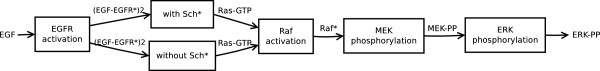
**Building blocks of the EGFR reaction network.** Each module communicates with the adjacent modules through few species only. Additionally, with the exception of the first module, all the others are double, one external (i.e., outside the cell surface) and one internal.

The EGFR model is a well-studied system describing signaling phenomena of (mammalian) cells [29-31]. As its name suggests, EGFR regulates cell growth, survival, proliferation and differentiation and plays a complex and crucial role during embryonic development and in tumor progression [60,61]. In this paper, we study the reaction network model for the dynamics of EGFR developed by Schoeberl et al. [31] which consists of 94 species and 207 reactions. Figure 6 presents the EGFR reaction network in an abstract level. Initially, the extracellular binding of EGF with the EGF receptors induce receptor dimerization. Then, two principal pathways, Shc-dependent and Sch-independend, are initiated leading to activation of Ras-GTP. Subsequently phosphorylation of MEK kinase through the activation of Raf kinase occurs leading to the phosphorylation of ERK kinase which regulates several proteins and nuclear transcription factors inside the cell. The detailed graphical description of the reaction network can be found in the Figures one & two of supplementary information in [31]. For completeness, all the reactions along with their rates are provided in the Additional file 3 of this publication.

The propensity functions for the reactions *R*_1_, …, *R*_97_, *R*_100_, …, *R*_207_ of the EGFR network are written in the general form

(29) aj(x)=kjxAjαjxBjβj,j=1,…,97,100,…,207

with the exception of reaction pair *R*_98_, *R*_99_ where their propensity functions are governed by the Michaelis-Menten kinetics

(30) $$a_{j}(\text{x})=V_{\max}{\text{x}}_{A_{j}}/(K_{m}+{\text{x}}_{A_{j}}),\;j=98,99$$

where **x** is the current state of the reaction system while *A*_*j*_ corresponds to the reacting species. The parameter vector contains all the reaction constants,

$$\theta =\;{\left[\;k_{1},\ldots ,k_{97},V_{\max},K_{m},k_{100},\ldots ,k_{207}\right]}^{T},$$

with all values provided in the Additional file 3. Due to the specific values of the reaction constants as well as the initial population of the species (see Table 3), the firing rates between reactions differ by many orders of magnitude giving rise to a highly stiff network. Therefore, even though there are some stochastic implementations, [27], here for the purposes of RER and FIM calculations, we adopt the mean-field approximation discussed in the accelerated estimators subsection. We solve the derived system of ODEs with Matlab’s routine ode15s and compute the FIM at the steady state regime which corresponds to the time interval [500,700]. The completion of the internalization process needs about 500 seconds. It should be noted here that even though the simulation of the EGFR is performed utilizing a deterministic approximation model, the computed pathwise FIM has been derived from the *stochastic* network, i.e., (13). This approximation is expected to be valid in the sense of (19) due to the large populations considered here. Overall, the computed FIM is a sparse matrix and measures efficiently the sensitivities of the stochastic model in a gradient-free manner.

**Table 3:** Initial population of the species for the EGFR network

**EGF**	**EGFR**	**GAP**	**Grb2**	**Sos**	**Ras-GDP**	**Shc**
4.98e10	5e4	1.2e4	5.1e4	6.63e4	1.14e7	1.01e6
Raf	Phosphatase 1	Phosphatase 2	Phosphatase 3	MEK	ERK	Pxrot
4e4	4e4	4e4	1e6	2.2e7	2.1e7	8.1e4

**Figure 7:**
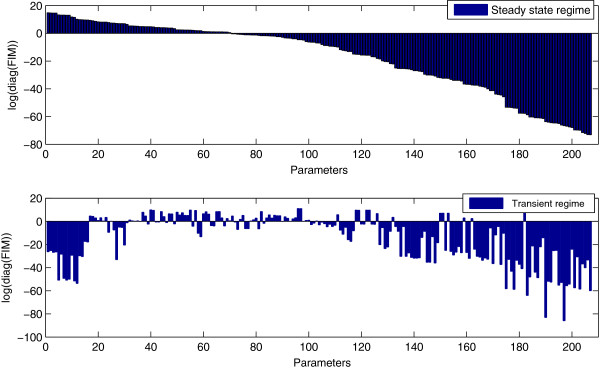
**Diagonal elements of the FIM computed at the steady state regime (upper plot) and at the transient regime (lower plot).** Note the changes in sensitivity and consequently the parameter identifiability. The parameter sensitivities differ by orders of magnitude.

The upper plot of Figure 7 shows the diagonal elements of the FIM in descending order computed at the steady state regime. We report our results in the format of Figure 7 in order to be able to accommodate the large number of parameters in the model. The *k*-th diagonal element of the FIM corresponds to RER where the perturbation takes place only to the *k*-th parameter (see (5)). Figure 7 (upper plot) in conjunction with Table S1 of the Additional file 4 fully describe the (local) sensitivities of the reaction network. Table S1 in Additional file 4 presents the reaction constants ordered from the most sensitive to the least sensitive parameter. Moreover, the FIM is diagonal –except a small 2×2 block associated with the Michaelis-Menten reactions– therefore the diagonal elements correspond to the eigenvalues of the FIM. The sensitivity analysis depicted in Figure 7, demonstrates that most model parameters allow for a vast range of perturbations without affecting the dynamics. Furthermore, this robustness to variations in most parameters was also reported in the original, fully deterministic EGFR model in [31]. This is a feature shared by many multi-parameter models in systems biology and which is known as “sloppiness”, [38]. Our methodology can easily demonstrate such properties in stochastic dynamics, as we can readily see in Figure 7, even if the models include a large number of parameters.

The previous discussion refers to the analysis of the EGFR model to the steady state regime. On the other hand, EGFR is a signaling model whose transient regime, in addition to the steady state, is of great interest. As discussed in Remark 3, we can justify the application of the RER and FIM sensitivity analysis in the transient regime. Therefore, we compute the proposed FIM at the time interval [0,10], using (22). The lower plot of Figure 7 shows the diagonal elements of the pathwise FIM in the transient regime while keeping the ordering of the parameters unchanged from the upper, steady state plot. The parameter sensitivity ordering is completely different meaning that the sensitivities are time-dependent in the transient regime. For instance, the most sensitive parameters in the stationary regime correspond to the final products of the reaction network, however, in the time interval [0,10] these species have not been produced yet resulting to insensitive reaction constants. In terms of parameter identification and estimation, the time-dependent sensitivities imply that in order to extract the maximum information content from the experimental data, we have to estimate the parameters drawing samples from different time intervals. These time intervals should be defined based on the respective sensitivity indices and selected in order to maximize the identifiability for each set of parameters.

**Figure 8:**
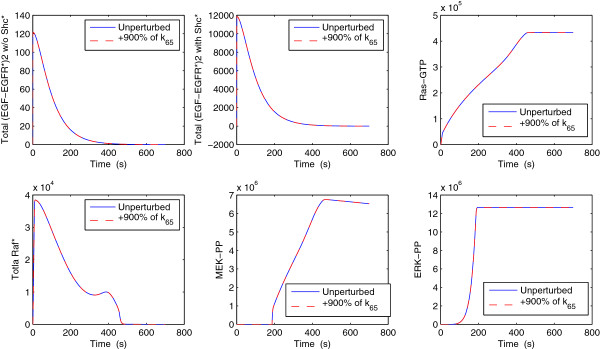
**Time-dependent concentration of various species of the EGFR network either for the unperturbed parameter vector (solid blue lines) or for the perturbed one (dashed red lines).** The 140-th most sensitive parameter (*k*_65_) is ten-fold increased and the species concentrations are not affected. For the least sensitive parameters such as *k*_65_, we rigorously know from the Pinsker inequality (9) that they should not alter the concentration values or any other observable even when they are heavily perturbed.

Finally, the Pinsker inequality (9) suggests that insensitive parameters can be perturbed, even significantly, without affecting species concentrations or other observable. As an illustration of this fact, we present in Figure 8 the concentrations of various critical species of the EGFR model when the 140-th (*k*_65_) most sensitive parameter is perturbed (see Table S1 in Additional file 4). The rate constant *k*_65_ corresponds to a reaction of the Shc-dependent pathway module. Solid blue lines correspond to the unperturbed parameter case while the dashed red lines correspond to the perturbed case where the perturbation is a multiplication by a factor of ten of *k*_65_. We present the total number of (EGF-EGFR*)2 binding species without Sch* (top, left panel) and with Sch* (top, middle panel) as well as Ras-GTP (top, right panel), total activated Raf or total Raf* (low, left panel), doubly phosphorated MEK or MEK-PP (low, middle panel) and doubly phosphorated ERK or ERK-PP. These species are important for the understanding of the system since the different modules of the EGFR reaction network communicate through them (see Figure 6). It is evident from Figure 8 that the various species concentrations remain unchanged to perturbations of the insensitive parameter *k*_65_ as it was expected from (9). Moreover, we notice that although the average populations become large, which implies that the maximum norm in (9) is also large, we still obtained robust results regarding *k*_65_’s parameter sensitivity.

## Conclusions

In this paper, we applied and extended a recently proposed parametric sensitivity analysis methodology to complex stochastic reaction networks. This sensitivity analysis approach is based on the quantification of information loss along different parameter perturbations between time-series distributions. This is achieved by employing the Relative Entropy Rate, which is directly computable from the propensity functions. A key aspect of the method is that we can derive rigorously an associated Fisher Information Matrix on path-space, which in turn constitutes a gradient-free approach for parametric sensitivity analysis; as such it provides a significant advantage in stochastic systems with a large number of parameters. We demonstrated that the structure of the pathwise FIM revealed hidden parameter interdependencies between the reactions. The block-diagonal structure of the FIM highlighted the sparsity of the matrix which resulted in further improvements in the computational efficiency of the proposed method. Therefore, parametric sensitivity analysis for high-dimensional stochastic reaction systems becomes tractable since it is well-known that in high dimensional stochastic systems sensitivity analysis techniques can involve estimators of very high variance, e.g. in finite difference methods and their recently proposed variants, which can present an overwhelming computational cost. Additionally, we proposed the use of multiscale numerical approximations of stochastic reaction networks in order to derive efficient statistical estimators for the FIM and implemented one such approximation (mean-field) in a high-dimensional system.

The proposed pathwise sensitivity analysis method is tested and validated on three biological systems: (a) a simple protein production/degradation model where explicit solutions are available, (b) the p53 reaction network where quasi-steady stochastic oscillations of the concentrations are observed and where multiscale stochastic approximations break down due to the persistent oscillations between low and high populations, and (c) a stochastic EGFR model which is an example of a high-dimensional reaction network with more than 200 reactions and a corresponding number of parameters. In the EGFR reaction network, we combined the proposed pathwise FIM which has been derived from the stochastic network and the mean-field approximation which is used for the efficient estimation of the pathwise FIM. Moreover, our earlier rigorous analysis for the steady state regime [19] suggests suitable extensions in the transient regime which were tested and validated for the EGFR model. We will present the full rigorous theory in an upcoming publication.

Finally, we note that the relation between RER and various observables is not straightforward. However, we note that the path distribution contains all information regarding the process including the steady state and all time-dependent observables: practically, our proposed sensitivity analysis represents a “conservative” sensitivity estimate in the sense that insensitive directions for the relative entropy on path-space will yield insensitive directions for every observable. This latter statement can be justified mathematically through the Pinsker inequality (9) which was tested in the examples considered here. Based on these observations, the proposed sensitivity analysis methods can be deployed in complementary fashion with existing sensitivity analysis tools, as it can be used to narrow down the most sensitive directions in a system.

## Competing interests

The authors declare that they have no competing interests.

## Authors’ contributions

MAK and YP conceived the proposed sensitivity analysis methodology. YP conducted the numerical experiments. MAK and DGV designed and supervised the conducted research. All authors contributed to the preparation of the manuscript. All authors read and approved the final manuscript.

## Supplementary Material

Additional file 1 The detailed derivation of relative entropy rate and the associated Fisher information matrix. Click here for file

Additional file 2 The calculation of equilibrium and pathwise FIMs for the protein production/degradation model. Click here for file

Additional file 3 This file contains in plain text the reactions and the reaction constants of the EGFR model. Click here for file

Additional file 4 The ordering of the parameter sensitivities for the EGFR model. Click here for file
