# Supplementary material for: Parametric sensitivity analysis for biochemical reaction networks based on pathwise information theory
Source: BMC Bioinformatics. 2013 Oct 22;14:311. doi: 10.1186/1471-2105-14-311 (PMC4015035; doi:10.1186/1471-2105-14-311)
Supplement: Additional file 1 — The detailed derivation of relative entropy rate and the associated Fisher information matrix. [file 1471-2105-14-311-S1.pdf]

Derivation of the Relative Entropy Rate and the pathwise Fisher Information Matrix

Yannis Pantazis, Markos A. Katsoulakis and Dionisios G. Vlachos

In order to derive the explicit formula for the relative entropy rate, we slightly adapt the notation for the propensity functions. We define the transition rate function as

$$a(\mathbf{X}_{s-}, \mathbf{X}_s) = \begin{cases} a_j(\mathbf{X}_{s-}) & \text{if } \mathbf{X}_s = \mathbf{X}_{s-} + \nu_{\mathbf{j}} \\ 0 & \text{otherwise} \end{cases} .$$

The new notation implies that when a jump occurs at time instant s, $\mathbf{X}_{s-}$ denotes the state of the process just before the jump while $\mathbf{X}_s$ denotes the new state of the process after the jump.

Then, the Radon-Nikodym derivative of the path distribution $Q^{\theta}_{[0,T]}$ with respect to the path distribution $Q^{\theta+\epsilon}_{[0,T]}$ has an explicit formula known also as Girsanov formula [1, 2]

$$\frac{dQ^{\theta}_{[0,T]}}{dQ^{\theta+\epsilon}_{[0,T]}}(\{\mathbf{X}_t\}) = \frac{\mu^{\theta}(\mathbf{X}_0)}{\mu^{\theta+\epsilon}(\mathbf{X}_0)} \exp\left\{ \int_0^T \log \frac{a^{\theta}(\mathbf{X}_{s-}, \mathbf{X}_s)}{a^{\theta+\epsilon}(\mathbf{X}_{s-}, \mathbf{X}_s)} dN_s - \int_0^T [a_0^{\theta}(\mathbf{X}_s) - a_0^{\theta+\epsilon}(\mathbf{X}_s)]\, ds \right\}$$

where μ^{θ} (reps. $\mu^{\theta+\epsilon}$) is the stationary distributions of $\{\mathbf{X}_t\}_{t\in\mathbb{R}_+}$ (resp. $\{\widetilde{\mathbf{X}}_t\}_{t\in\mathbb{R}_+}$) while N_s is the counting (of the jumps) measure. The explicit formula for the RER was first given by Dumitrescu [3] for finite state spaces. Here, we reproduce the derivation for the sake of completeness. Using the Girsanov formula, the relative entropy (eq. (2) in the main text) is rewritten as

$$\begin{aligned} \mathcal{R}\left(Q^{\theta}_{[0,T]} \,|\, Q^{\theta+\epsilon}_{[0,T]}\right) &= \mathbb{E}_{Q^{\theta}_{[0,T]}}\left[\log \frac{\mu^{\theta}(\mathbf{X}_0)}{\mu^{\theta+\epsilon}(\mathbf{X}_0)} + \int_0^T \log \frac{a^{\theta}(\mathbf{X}_{s-}, \mathbf{X}_s)}{a^{\theta+\epsilon}(\mathbf{X}_{s-}, \mathbf{X}_s)}\, dN_s - \int_0^T [a_0^{\theta}(\mathbf{X}_s) - a_0^{\theta+\epsilon}(\mathbf{X}_s)]\, ds \right] \\ &= \mathbb{E}_{Q^{\theta}_{[0,T]}}\left[\int_0^T \log \frac{a^{\theta}(\mathbf{X}_{s-}, \mathbf{X}_s)}{a^{\theta+\epsilon}(\mathbf{X}_{s-}, \mathbf{X}_s)}\, dN_s \right] - \mathbb{E}_{Q^{\theta}_{[0,T]}}\left[\int_0^T [a_0^{\theta}(\mathbf{X}_s) - a_0^{\theta+\epsilon}(\mathbf{X}_s)]\, ds \right] + \mathbb{E}_{Q^{\theta}_{[0,T]}}\left[\log \frac{\mu^{\theta}(\mathbf{X}_0)}{\mu^{\theta+\epsilon}(\mathbf{X}_0)} \right] . \end{aligned}$$

Exploiting the fact that the process $M_t := N_t - \int_0^t a_0^{\theta}(\mathbf{X}_s) ds$ is a martingale, we have that

$$\mathbb{E}_{Q^{\theta}_{[0,T]}}\left[\int_0^T \log \frac{a^{\theta}(\mathbf{X}_{s-}, \mathbf{X}_s)}{a^{\theta+\epsilon}(\mathbf{X}_{s-}, \mathbf{X}_s)}\, dN_s \right] = \mathbb{E}_{Q^{\theta}_{[0,T]}}\left[\int_0^T a_0^{\theta}(\mathbf{X}_{s-}) \log \frac{a^{\theta}(\mathbf{X}_{s-}, \mathbf{X}_s)}{a^{\theta+\epsilon}(\mathbf{X}_{s-}, \mathbf{X}_s)}\, ds \right] .$$

Moreover, changing the order of the integrals and due to the stationarity of the process $\{\mathbf{X}_t\}_{t\in\mathbb{R}_+}$, the relative entropy is simplified to the following:

$$\begin{aligned} \mathcal{R}\left(Q^{\theta}_{[0,T]} \,|\, Q^{\theta+\epsilon}_{[0,T]}\right) &= \int_0^T \mathbb{E}_{\mu^{\theta}}\left[\sum_{\mathbf{x}'\in E} a_0^{\theta}(\mathbf{x}) \frac{a^{\theta}(\mathbf{x}, \mathbf{x}')}{a_0^{\theta}(\mathbf{x})} \log \frac{a^{\theta}(\mathbf{x}, \mathbf{x}')}{a^{\theta+\epsilon}(\mathbf{x}, \mathbf{x}')} \right] ds - \int_0^T \mathbb{E}_{\mu^{\theta}}\left[a_0^{\theta}(\mathbf{x}) - a_0^{\theta+\epsilon}(\mathbf{x}) \right] ds + \mathbb{E}_{\mu^{\theta}}\left[\log \frac{\mu^{\theta}(\mathbf{x})}{\mu^{\theta+\epsilon}(\mathbf{x})} \right] \\ &= T\mathcal{H}\left(Q^{\theta} \,|\, Q^{\theta+\epsilon}\right) + \mathcal{R}\left(\mu^{\theta} \,|\, \mu^{\theta+\epsilon}\right) , \end{aligned}$$

where

$$\mathcal{H}\left(Q^\theta \,|\, Q^{\theta+\epsilon}\right) = \mathbb{E}_{\mu^\theta}\Big[\sum_{\mathbf{x}'\in E} a^\theta(\mathbf{x},\mathbf{x}')\log\frac{a^\theta(\mathbf{x},\mathbf{x}')}{a^{\theta+\epsilon}(\mathbf{x},\mathbf{x}')} - \left(a_0^\theta(\mathbf{x}) - a_0^{\theta+\epsilon}(\mathbf{x})\right)\Big]$$

$$= \mathbb{E}_{\mu^\theta}\Big[\sum_{j=1}^M a_j^\theta(\mathbf{x})\log\frac{a_j^\theta(\mathbf{x})}{a_j^{\theta+\epsilon}(\mathbf{x})} - \left(a_0^\theta(\mathbf{x}) - a_0^{\theta+\epsilon}(\mathbf{x})\right)\Big]$$

is the relative entropy rate.

The pathwise Fisher Information Matrix is derived next from the RER. Defining the rate difference $\delta a_j(\mathbf{x}) = a_j^{\theta+\epsilon}(\mathbf{x}) - a_j^\theta(\mathbf{x})$, the relative entropy rate of $Q_{[0,T]}^\theta$ w.r.t. $Q_{[0,T]}^{\theta+\epsilon}$ is equal to

$$\mathcal{H}\left(Q^\theta \,|\, Q^{\theta+\epsilon}\right) = -\sum_{\mathbf{x}\in E}\mu^\theta(\mathbf{x})\sum_{j=1}^M a_j^\theta(\mathbf{x})\log\left(1+\frac{\delta a_j(\mathbf{x})}{a_j^\theta(\mathbf{x})}\right) + \sum_{\mathbf{x}\in E}\mu^\theta(\mathbf{x})\sum_{j=1}^M \delta a_j(\mathbf{x})$$

$$= -\sum_{\mathbf{x}\in E}\mu^\theta(\mathbf{x})\sum_{j=1}^M\Big[\delta a_j(\mathbf{x}) - \frac{1}{2}\frac{\delta a_j(\mathbf{x})^2}{a_j^\theta(\mathbf{x})} + O(|\delta a_j(\mathbf{x})|^3)\Big] + \sum_{\mathbf{x}\in E}\mu^\theta(\mathbf{x})\sum_{j=1}^M \delta a_j(\mathbf{x})$$

$$= \frac{1}{2}\sum_{\mathbf{x}\in E}\mu^\theta(\mathbf{x})\sum_{j=1}^M\frac{\delta a_j(\mathbf{x})^2}{a_j^\theta(\mathbf{x})} + O(|\delta a_j(\mathbf{x})|^3)\ .$$

Under a (checkable) differentiability assumption on the transition rates in a neighborhood of parameter vector θ, which is a checkable hypothesis, a Taylor series expansion of $\delta a_j(\mathbf{x}) = \epsilon^T\nabla_\theta a_j^\theta(\mathbf{x}) + O(|\epsilon|^2)$ results in

$$\mathcal{H}\left(Q^\theta \,|\, Q^{\theta+\epsilon}\right) = \frac{1}{2}\sum_{\mathbf{x}\in E}\mu^\theta(\mathbf{x})\sum_{j=1}^M\frac{\left(\epsilon^T\nabla_\theta a_j^\theta(\mathbf{x})\right)^2}{a_j^\theta(\mathbf{x})} + O(|\epsilon|^3)$$

$$= \frac{1}{2}\epsilon^T\Big(\sum_{\mathbf{x}\in E}\mu^\theta(\mathbf{x})\sum_{j=1}^M a_j^\theta(\mathbf{x})\nabla_\theta\log a_j^\theta(\mathbf{x})\nabla_\theta\log a_j^\theta(\mathbf{x})^T\Big)\epsilon + O(|\epsilon|^3)$$

$$= \frac{1}{2}\epsilon^T\mathbf{F}_\mathcal{H}(Q^\theta)\epsilon + O(|\epsilon|^3)\ ,$$

where

$$\mathbf{F}_\mathcal{H}(Q^\theta) := \mathbb{E}_{\mu^\theta}\left[\sum_{j=1}^M a_j^\theta(\mathbf{x})\nabla_\theta\log a_j^\theta(\mathbf{x})\nabla_\theta\log a_j^\theta(\mathbf{x})^T\right]$$

is the pathwise Fisher information matrix.

References

[1] R. S. Liptser and A. N. Shiryaev. *Statistics of Random Processes: I & II.* Springer, 1977.

[2] C. Kipnis and C. Landim. *Scaling Limits of Interacting Particle Systems.* Springer-Verlag, 1999.

[3] M. E. Dumitrescu. Some informational properties of markov pure-jump processes. *C. P. Matematiky*, 113:429–434, 1988.
