# Supplementary material for: Parametric sensitivity analysis for biochemical reaction networks based on pathwise information theory
Source: BMC Bioinformatics. 2013 Oct 22;14:311. doi: 10.1186/1471-2105-14-311 (PMC4015035; doi:10.1186/1471-2105-14-311)
Supplement: Additional file 2 — The calculation of equilibrium and pathwise FIMs for the protein production/degradation model. [file 1471-2105-14-311-S2.pdf]

Derivation of the equilibrium and pathwise Fisher Information Matrices for a simple birth/death model

Yannis Pantazis, Markos A. Katsoulakis and Dionisios G. Vlachos

The single-species birth/death reaction network is defined by the reaction pair

$$\emptyset \underset{k_2}{\overset{k_1}{\rightleftarrows}} X \ . \tag{1}$$

The two propensity functions are $a_1(x) = k_1$ for the birth event and $a_2(x) = k_2 x$ for the death event. The equilibrium distribution, $\mu^\theta(x)$, of this simple network where $\theta = [k_1, k_2]^T$ is Poisson with parameter $\frac{k_1}{k_2}$, i.e.,

$$\mu^\theta(x) = \left(\frac{k_1}{k_2}\right)^x \frac{e^{-\frac{k_1}{k_2}}}{x!} \ . \tag{2}$$

The pathwise FIM in the logarithmic scale equals to

$$\mathbf{F}_\mathcal{H}(Q^{\log\theta}) := \begin{bmatrix} \mathbb{E}_{\mu^\theta}[a_1(x)] & 0 \\ 0 & \mathbb{E}_{\mu^\theta}[a_2(x)] \end{bmatrix} = \begin{bmatrix} k_1\mathbb{E}_{\mu^\theta}[1] & 0 \\ 0 & k_2\mathbb{E}_{\mu^\theta}[x] \end{bmatrix} = \begin{bmatrix} k_1 & 0 \\ 0 & k_1 \end{bmatrix} \tag{3}$$

The equilibrium FIM is given in logarithmic scale by

$$\begin{aligned} \mathbf{F}_\mathcal{R}(\mu^{\log\theta}) &:= \begin{bmatrix} k_1^2\mathbb{E}_{\mu^\theta}[(\partial_{k_1}\log(\mu^\theta(x)))^2] & k_1k_2\mathbb{E}_{\mu^\theta}[\partial_{k_1}\log(\mu^\theta(x))\partial_{k_2}\log(\mu^\theta(x))] \\ k_1k_2\mathbb{E}_{\mu^\theta}[\partial_{k_1}\log(\mu^\theta(x))\partial_{k_2}\log(\mu^\theta(x))] & k_2^2\mathbb{E}_{\mu^\theta}[(\partial_{k_2}\log(\mu^\theta(x)))^2] \end{bmatrix} \\ &= \begin{bmatrix} \mathbb{E}_{\mu^\theta}[(x-\frac{k_1}{k_2})^2] & \mathbb{E}_{\mu^\theta}[(x-\frac{k_1}{k_2})(-x+\frac{k_1}{k_2})] \\ \mathbb{E}_{\mu^\theta}[(x-\frac{k_1}{k_2})(-x+\frac{k_1}{k_2})] & \mathbb{E}_{\mu^\theta}[(-x+\frac{k_1}{k_2})^2] \end{bmatrix} \\ &= \begin{bmatrix} \frac{k_1}{k_2} & -\frac{k_1}{k_2} \\ -\frac{k_1}{k_2} & \frac{k_1}{k_2} \end{bmatrix} \end{aligned} \tag{4}$$
