# Supplementary material for: Parametric sensitivity analysis for biochemical reaction networks based on pathwise information theory
Source: BMC Bioinformatics. 2013 Oct 22;14:311. doi: 10.1186/1471-2105-14-311 (PMC4015035; doi:10.1186/1471-2105-14-311)
Supplement: Additional file 4 — The ordering of the parameter sensitivities for the EGFR model. [file 1471-2105-14-311-S4.pdf]

EGFR parameter ordering table

Yannis Pantazis, Markos A. Katsoulakis and Dionisios G. Vlachos

July 1, 2013

Table 1: Ordering of the reaction rate constants based on their sensitivity index computed at the stationary regime. From left to right and from up to down.

k_{162}	k_{156}	k_{166}	k_{160}	k_{164}	k_{163}	k_{158}	k_{161}	k_{167}	k_{157}	k_{165}	k_{159}	k_{140}	k_{141}	k_{138}
k_{139}	k_{68}	k_{69}	k_{80}	k_{122}	k_{76}	k_{125}	k_{72}	k_{81}	k_{85}	k_{84}	k_{143}	k_{134}	k_{137}	k_{142}
k_{123}	k_{95}	k_{94}	k_{36}	k_{89}	k_{37}	k_{5}	k_{6}	k_{88}	k_{144}	k_{145}	k_{111}	k_{110}	k_{97}	k_{96}
k_{40}	k_{132}	k_{136}	k_{77}	k_{3}	k_{4}	k_{148}	k_{128}	k_{131}	k_{66}	k_{45}	k_{67}	k_{135}	k_{44}	k_{120}
k_{78}	k_{124}	k_{21}	k_{20}	k_{74}	k_{70}	k_{146}	k_{49}	k_{147}	K_m	k_{121}	k_{12}	k_{48}	k_{79}	k_{83}
k_{19}	k_{18}	k_{149}	k_{133}	k_{82}	k_{176}	k_{1}	k_{22}	k_{25}	k_{87}	k_{206}	k_{34}	k_{35}	k_{200}	k_{129}
k_{38}	k_{86}	k_{207}	k_{202}	k_{75}	k_{126}	k_{130}	k_{201}	k_{43}	k_{47}	k_{203}	k_{194}	k_{189}	k_{42}	k_{185}
k_{46}	k_{195}	k_{191}	k_{190}	k_{186}	k_{127}	k_{192}	k_{2}	k_{179}	k_{17}	k_{16}	k_{180}	k_{168}	k_{172}	k_{152}
k_{155}	k_{150}	k_{154}	k_{170}	k_{173}	k_{169}	k_{11}	k_{171}	k_{175}	k_{174}	k_{153}	k_{151}	k_{118}	k_{52}	k_{92}
k_{56}	k_{109}	k_{108}	k_{119}	k_{65}	k_{64}	$V_{\max}$	k_{73}	k_{53}	k_{32}	k_{33}	k_{103}	k_{102}	k_{41}	k_{116}
k_{50}	k_{90}	k_{54}	k_{117}	k_{205}	k_{63}	k_{62}	k_{198}	k_{51}	k_{93}	k_{199}	k_{101}	k_{30}	k_{31}	k_{100}
k_{193}	k_{187}	k_{184}	k_{71}	k_{188}	k_{91}	k_{181}	k_{178}	k_{39}	k_{61}	k_{57}	k_{10}	k_{114}	k_{106}	k_{60}
k_{24}	k_{13}	k_{29}	k_{59}	k_{55}	k_{28}	k_{112}	k_{104}	k_{23}	k_{204}	k_{196}	k_{107}	k_{58}	k_{14}	k_{115}
k_{27}	k_{197}	k_{9}	k_{7}	k_{26}	k_{183}	k_{182}	k_{8}	k_{105}	k_{113}	k_{15}	k_{177}	—	—	—
